# The association between shift work and hyperuricemia in steelmaking male workers

**DOI:** 10.1186/s40557-014-0042-z

**Published:** 2014-11-04

**Authors:** Jae-Seok Oh, Won-Jun Choi, Min-Kee Lee, Sung-Woo Han, Seung-Ho Song, Jong-Wan Yun, Sang-Hwan Han

**Affiliations:** Department of Occupational and Environmental Medicine, Gachon University Gil Medical Center, 72-39, Namdong-daero 239beon-gil, Namdong-gu, Incheon, Korea

**Keywords:** Hyperuricemia, Shift work, Job schedule

## Abstract

**Objectives:**

The aim of this study was to examine the association between shift work and hyperuricemia among steel company workers.

**Methods:**

We examined 1,029 male workers at a Korean steel company between June 6 and June 28, 2013. We conducted anthropometric measurements, questionnaire surveys, and blood tests. Hyperuricemia was defined as a serum uric acid concentration of ≥7.0 mg/dL. Logistic regression analyses were performed. In the full model, analysis was adjusted for covariates including age, body mass index, lifestyle factors, and comorbidities. The odds ratios (ORs) and 95% confidence intervals (95% CIs) were calculated for all models.

**Results:**

The participants included 276 daytime workers and 753 shift workers. Among daytime workers, 72 (26.1%) individuals had hyperuricemia, as did 282 (37.5%) individuals among shift workers (p <0.001). There was a statistically significant association between shift work and hyperuricemia. In the unadjusted model, the OR of shift work was 1.70 (95% CI 1.25-2.31) for hyperuricemia. In the full model, the OR of shift work was also statistically significant after adjustment for covariates (OR 1.41, 95% CI 1.02-1.96).

**Conclusions:**

Among male steel workers, a significant association between shift work and hyperuricemia was observed.

## Introduction

The International Labour Office (International Labour Organization, 1990a) defines working in shifts as ‘a method of organization of working time in which workers succeed one another at the workplace so that the establishment can operate longer than the hours of work of individual workers’ [1]. According to the 5th European Working Conditions Survey that was conducted in 34 European countries and published in 2010, 17% of all workers were engaged in shift work. In 27 European Union countries, 23% of male workers and 14% of female workers worked night shifts [2]. In Korea, according to the 2010 Survey Report on Labor Conditions by Employment Type among the total working population, approximately 11%, or 1,270,000 workers, were engaged in shift work.

Shift work disrupts the circadian rhythms of workers, and may cause various problems [3]. The impact of shift work on health has been actively studied [4]. Various studies have examined the impact of shift work on cardiovascular diseases [5,6], elevated serum cholesterol level [7], gastrointestinal disorders [8], cerebrovascular diseases [9], reproductive system disorders [10], mental illness, such as depression [11], bone mineral density [12], and others. In addition, some studies have examined the correlation of shift work with various cancers, including breast [13,14] and prostate cancers [15]. In 2010, the International Agency for Research on Cancer designated shift work as a Group 2A carcinogen, meaning it may cause cancer.

The effects of shift work, including night shifts, on health have received considerable attention. In order to protect the health of night shift workers, the Korean government introduced special medical examinations for night shift workers in January 2014.

Increased production of uric acid or decreased excretion of uric acid can be manifested as hyperuricemia. Uric acid can be accumulated in body tissues asymptomatically or presented as gout. Recent studies suggest that hyperuricemia is a risk factor for renal and cardiovascular disease [16–18]. It has been suggested that the elevation of serum uric acid is caused by numerous factors including genetic factors [19], renal diseases [20], lifestyle factors such as alcohol intake, diet, and obesity [21–23] or the use of diuretics [24]. However few studies have reported the effect of occupational factors that may elevate serum uric acid. In this study, we investigated the association between hyperuricemia and shift work among steel company workers.

## Materials and methods

### Study subjects

We conducted medical examinations of steel company workers in Incheon, Korea, between June 6 and June 28, 2013. In the company surveyed, the steel-making process is continuously carried out. Therefore, its workers perform in a four-crew-three-shift system; almost 75% of workers perform shift work. A total of 1,156 production workers were examined, with the vast majority, 1,132 (97.9%), being male. Among the male production workers, we excluded 103 individuals who provided insufficient answers in the questionnaire surveys or who did not complete anthropometric measurements or blood tests. Therefore, a total of 1,029 workers were finally selected as research subjects.

### Questionnaire surveys, anthropometric measurements, and blood tests

Through self-administered questionnaires, working conditions and experiences, along with lifestyle patterns, were investigated. Questions regarding working conditions sought information on office work/non-office work, shift work/daytime work, and work department. The workers who worked regular working hours (e.g., 9 AM to 5 PM) were classified into the ‘daytime work group’ and the remainder into the ‘shift work group’. Lifestyle questions sought information on smoking status, alcohol consumption, physical activity, and others; another section dealt with the medical history of hypertension, diabetes mellitus, hyperlipidemia, and current medications. Smoking status was divided into three groups: current smokers, former smokers, and never smokers. Alcohol consumption was measured by frequency of consumption (days/week) and amount consumed per session (glasses/time). Those who drank more than twice a week and had >7 glasses of alcohol in each session were designated as the high drinker group. Physical activity was examined by frequency (days/week), where those participating in strenuous physical activity >3 times/week or participating in moderate physical activity >5 times/week were classified into the active group.

Anthropometric measurements were taken. After participants had fasted for more than 8 hours, venous blood sampling was performed. Fasting levels of plasma glucose, total cholesterol, triglycerides, high-density lipoprotein (HDL) cholesterol, aspartate aminotransferase (AST), alanine aminotransferase (ALT), gamma-glutamyl transpeptidase (γ-GTP), blood urea nitrogen, creatinine, and serum uric acid were measured. Serum uric acid concentration was measured using the Uricase EMST method (Hitachi 747 Automatic Analyzer, Hitachi, Tokyo, Japan).

The physiochemical definition of hyperuricemia may be considered to be a serum uric acid concentration of 7.0 mg/dL or above, as measured by the specific uricase method. This represents the solubility limit of urate in plasma at 37°C. Uric acid levels above 7.0 mg/dL result in supersaturated solutions that are prone to crystal formation [25]. Based on previous studies, we defined hyperuricemia as a serum uric acid level ≥7.0 mg/dL [19,26–30]. Hypertension was defined as current hypertension medication use, or a systolic blood pressure ≥140 mmHg, or a diastolic blood pressure ≥90 mmHg. Diabetes was defined as currently being treated for diabetes or fasting blood glucose levels ≥126 mg/dL. Individuals whose LDL level was higher than 160 mg/dL or who reported that they were currently being treated for hyperlipidemia were classified as having hyperlipidemia. Blood test results indicating liver enzyme levels of ≥50 IU/L (AST), ≥45 IU/L (ALT), or ≥78 IU/L (γ-GTP) identified participants with liver enzyme abnormality. For kidney function, the estimated glomerular filtration rate (eGFR) value was calculated using the serum creatinine level. Individuals with an eGFR value of <90 mL/min/1.73 m^2^ were classified into the impaired renal function group [31].

### Statistical analysis

Statistical analyses were performed using PASW Statistics, Version 18.0 (SPSS Inc., Chicago, IL, USA). In the univariate analyses, *t*-tests were used for continuous variables and chi-square tests were used for the categorical variables. To determine the association between hyperuricemia and shift work, logistic regression analyses were used. The unadjusted model included only whether the subject was engaged in shift work or not. In the second model, analyses were adjusted for covariates including physical factors (age, BMI) and present health status (hypertension, diabetes, hyperuricemia, liver dysfunction, renal dysfunction). In the full model, lifestyle factors (smoking, alcohol consumption, physical activity) were additionally adjusted for in model 2. The odds ratio (OR) and 95% confidence interval (CI) were calculated for all models. Statistical significance was defined as a p-value of <0.05.

## Results

The general characteristics of the study participants are shown in Table 1. There were 753 (73.2%) shift workers and 276 (26.8%) daytime workers. Among the participants, 145 (14.1%) individuals were <20 years old, 326 (31.7%) were in their thirties, 192 (18.7%) were in their forties, and 366 (35.6%) were ≥50 years old. There were 570 (55.4%) individuals with a BMI under 25 kg/m^2^ and 354 (34.4%) individuals with hyperuricemia.

**Table 1:** **General characteristics of study subjects (n = 1029)**

		**n**	**%**
Total		1,029	100
Age (years)	~29	145	14.1
	30 ~ 39	326	31.7
	40 ~ 49	192	18.7
	50~	366	35.6
Body mass index	<25 kg/m^2^	570	55.4
	≥25 kg/m^2^	459	44.6
Job schedule	Shift work	753	73.2
	Daytime work	276	26.8
Hyperuricemia (≥7 mg/dl)	No	675	65.6
	Yes	354	34.4
Hypertension^*^	No	882	85.7
	Yes	147	14.3
Diabetes mellitus^†^	No	946	92.1
	Yes	83	7.9
Hyperlipidemia^‡^	No	738	71.7
	Yes	291	28.3
Liver enzyme abnormality^§^	No	811	78.8
	Yes	218	21.2

*Systolic BP ≥140 mmHg or diastolic BP ≥90 mmHg or currently taking medication for hypertension.

^†^Fasting blood glucose ≥126 mg/dl or currently taking medication for diabetes.

^‡^LDL ≥160 mg/dl or currently taking medication for hyperlipidemia.

^§^AST ≥ 50 IU/l or ALT 45 ≥ IU/l or γ-GTP ≥78 IU/l.

A comparison of the characteristics associated with shift work and daytime work is shown in Table 2. A total of 282 (37.5%) shift workers and 72 (26.1%) daytime workers had hyperuricemia; there was a significant difference between the incidence of hyperuricemia in the two groups (p < 0.001). Daytime workers’ age and work duration were significantly higher than those of shift workers. Hypertension, hyperlipidemia, impaired renal function also showed significantly higher prevalence in daytime workers. However, there were no significant differences in the incidence of diabetes or liver dysfunction between the shift workers and daytime workers. Daytime workers also do significantly more physical activity. However, no differences were observed between shift and daytime workers regarding smoking status and alcohol consumption.

**Table 2:** **Characteristics of study subjects by shift work**

		**Daytime work**		**Shift work**		**p-value**
		**(n = 276)**		**(n = 753)**		
		**n**	**%**	**n**	**%**	
Age (years)		46.3		40.2		<0.001^†^
BMI (kg/m^2^)		24.6		24.8		0.302^†^
Work duration (years)		20.1		13.9		<0.001^†^
Hyperuricemia (≥7 mg/dl)						
	Yes	72	26.1	282	37.5	**<0.001** ^*^
	No	204	73.9	471	62.5	
Hypertension						
	Yes	62	22.5	85	11.3	**<0.001** ^*^
	No	214	77.5	668	88.7	
Diabetes mellitus						
	Yes	27	9.8	56	7.4	0.221^*^
	No	249	90.2	697	92.6	
Hyperlipidemia						
	Yes	93	33.7	198	26.3	**0.020** ^*^
	No	183	66.3	555	73.7	
Liver enzyme abnormality						
	Yes	60	21.7	158	21.0	0.792^*^
	No	216	78.3	595	79.0	
Impaired renal function^‡^						
	Yes	144	52.2	290	38.5	**<0.001** ^*^
	No	132	47.8	463	61.5	
Smoking						
	Never-smoker or ex-smoker	153	55.4	403	53.5	0.585^*^
	Current smoker	123	44.6	350	46.5	
Sufficient physical activity						
	Yes^§^	100	36.2	224	29.7	**0.047** ^*^
	No	176	63.8	529	70.3	
Consumption of alcohol						
	Moderate drinker	181	65.6	516	68.5	0.370^*^
	High drinker^¶^	95	34.4	237	31.5	

*Calculated by chi-square test.

^†^Calculated by t-test.

^‡^EGFR <90 mL/min/1.73 m^2^.

^§^Vigorous-intensity physical activity more than three times per week or moderate-intensity physical activity more than five times per week.

^¶^drink alcohol more than two times per week and seven glasses per time.

The results of logistic regression analyses are presented in Table 3. There was a statistically significant association between shift work and hyperuricemia. The unadjusted OR of shift work for hyperuricemia was 1.70 (95% CI, 1.25–2.31). In the second model, shift work showed a statistically significant association with hyperuricemia (OR 1.41, 95% CI 1.01-1.95). In the full model, the association between shift work and hyperuricemia was still statistically significant after adjustment for possible confounders including comorbidities (OR 1.41, 95% CI 1.02-1.96). Age, BMI, diabetes, liver enzyme abnormality and impaired renal function were also statistically significant covariates in the full model.

**Table 3:** **Odds ratios for hyperuricemia in shift workers**

**Variable**	**Odds ratio (95% confidence interval)**		
	**Model 1**	**Model 2**	**Model 3**
Job schedule			
Daytime workers	Reference	Reference	Reference
Shift workers	**1.70 (1.25 ~ 2.31)**	**1.41 (1.01 ~ 1.95)**	**1.41 (1.02 ~ 1.96)**
Age (years)		0.95 (0.93 ~ 0.97)	0.95 (0.93 ~ 0.97)
Body mass index (kg/m^2^)		1.17 (1.11 ~ 1.24)	1.17 (1.11 ~ 1.24)
Hypertension		1.41 (0.92 ~ 2.15)	1.39 (0.91 ~ 2.13)
Diabetes		0.43 (0.23 ~ 0.80)	0.43 (0.23 ~ 0.81)
Hyperlipidemia		1.13 (0.83 ~ 1.54)	1.16 (0.85 ~ 1.58)
Liver enzyme abnormality		1.48 (1.06 ~ 2.08)	1.41 (1.00 ~ 1.99)
Impaired renal function		1.99 (1.35 ~ 2.93)	2.06 (1.40 ~ 3.03)
Smoking (versus non-smokers)			0.93 (0.71 ~ 1.24)
Physical activity (versus inactive)			1.33 (0.99 ~ 1.78)
Drinking (versus moderate drinker)			1.13 (0.84 ~ 1.53)

## Discussion

The aim of this study was to investigate the potential association between shift work and hyperuricemia. The results showed a high prevalence of hyperuricemia among shift workers compared with daytime workers. There was a statistically significant association between shift work and hyperuricemia after adjustment for covariates.

Important factors that may affect the health of shift workers include repeated changes between day shift and night shift, as well as the breakdown of circadian rhythms and sleep disturbances [32]. In fact, shift workers, compared to day workers, experience more insomnia [33], lower sleep quality, and consume more alcohol [34]. In this study, the shift and daytime worker groups did not show any significant difference in alcohol consumption (p = 0.370), but in terms of physical activity, the daytime worker group was significantly more active than the shift worker group (p = 0.047). This may be explained by irregular work hours, leading to irregular sleep hours, resulting in difficulties engaging in physical activities.

An association between shift work and serum uric acid concentration in railroad shift workers who repeatedly changed from day to night shift work has been reported [35]. In that study, when workers switched from day to night work their serum uric acid level increased significantly. In addition, research in Japanese telecommunication company workers established an association between shift work and hyperuricemia onset, particularly among males [36]. These authors explained their findings as the result of circadian rhythm changes that cause a lack of sleep and induce stress that leads to elevated serum uric acid concentration.

In humans, unlike other mammals, uric acid is the final breakdown product of purine metabolism and is excreted in the urine [37]. Uric acid can be accumulated in body tissues either asymptomatically or presented as gout. Recent studies suggest that hyperuricemia is a risk factor for renal and cardiovascular disease [16–18].

Although the underlying mechanism of how shift work leads to hyperuricemia has not been identified, recent studies suggest that circadian rhythm changes can lead to oxidant-antioxidant imbalances and cause oxidative stress. Such oxidative stress is thought to induce redox-dependent signaling in adipocytes, leading to the progression of hyperuricemia [38–40]. In a cross-sectional study involving nurses who did and did not engage in shift work, glutathione peroxidase activity was positively correlated to blood antioxidant concentrations, which were significantly higher in those involved in shift work. Furthermore, the results of another study on medical personnel who did and did not engage in shift work showed that shift work resulted in significantly higher oxidative stress indexes [39,40].

A laboratory study of murine adipocytes demonstrates that oxidative stress stimulates mature adipocytes. The enzyme NADPH oxidase involves the production of uric acid through an intracellular oxidation process [41]. In our study, we did not examine whether shift work could induce oxidative stress, making it difficult to determine whether the association between shift work and hyperuricemia progressed according to the previously reported mechanisms. However, various studies have reported that shift workers demonstrate significantly higher levels of stress than do non-shift workers [33,41], suggesting that there may be an association between shift work and hyperuricemia.

The inclusion of all company workers in the study in conjunction with the four-crew-three-shift system for the steel work process indicates that approximately 75% of the workers studied are engaged in shift work. There were significant differences in average age and work duration between shift workers and daytime workers. Previous studies also reported that similar distributions. Taking the nature of a steel-working company into consideration, people with longer tenure or in higher positions have increased opportunities to work as daytime workers. Furthermore, it is thought that production work is usually done by healthy workers, so we cannot exclude a healthy worker effect [42]. Shift workers include a higher proportion of production workers, and there is a significant difference in age between shift workers and daytime workers, thus we included age and other covariates in the three-step statistical model.

Personal lifestyle factors such as obesity and alcohol consumption are well-established risk factors for an increased serum uric acid level. Furthermore, recent epidemiological studies have reported that untreated hypertension, diabetes, hyperlipidemia, liver disease and/or renal disease are associated with serum uric acid level [30,43–46]. Unlike previous studies that only adjusted for personal lifestyle factors, our study included present health status covariates (hypertension, diabetes, hyperuricemia, liver dysfunction, renal dysfunction) in the full model that have been reported to be associated with serum uric acid level. Age, BMI, diabetes, liver enzyme abnormality, and renal dysfunction showed significant impact on the results of both our study and of previous studies. There is a negative association of age with uric acid level, in agreement with a previous longitudinal study that showed that younger men have higher weights than older men, which subsequently affects serum uric acid level [43]. In this study, weight tended to decrease as age increased, which could lead to a similar explanation for the association with uric acid level. BMI and liver enzyme abnormality showed positive associations with uric acid levels. To maintain normal uric acid levels, uric acid is removed from the body through the kidney. However, without appropriate renal function, uric acid cannot be removed from the body and hyperuricemia can occur. As continuously high glucose levels block reabsorption of uric acid from the proximal renal tubule, thus increase its excretion, diabetes (unlike pre-diabetes) is thought to show significant negative association with serum uric acid levels [44].

Previous studies have showed a positive association between hypertension and hyperuricemia, which is thought to be caused by renal insufficiency [45]. However, previous studies have focused on uncontrolled hypertension, while our study included workers who take medication to treat hypertension or who have been diagnosed with hypertension at a worker’s health examination. This may explain why our study identified a positive, but statistically insignificant trend towards an association between hypertension and hyperuricemia.

Hyperlipidemia and lifestyle factors (alcohol consumption, smoking, and physical activity) were not found to be significantly associated with hyperuricemia in our study. Previous studies which included the whole population showed that hyperlipidemia and alcohol consumption are positively associated with hyperuricemia. This association was especially prominent in the case of alcohol consumption, which showed a dose-response relationship with uric acid concentration [46]. As our study included only steel processing workers and classified them into high drinker and moderate drinker groups, further studies should be carried out in order to examine dose relations. A previous study reported a negative association between smoking and serum uric acid level [45]. However, in a different longitudinal study, no association was revealed [36]. Our study also could not find any significant relation between these variables, but additional study could be carried out to explore this further.

Our study examined the association between shift work and hyperuricemia in male steel company workers, limiting the analysis to those with similar types of work and work environments. Recently there has been increased interest in the prevention of cardiovascular diseases, with studies reporting hyperuricemia as a potential risk factor. Thus, the analysis and reporting of the relationship between shift work and hyperuricemia is important.

There are several limitations in this study. First, we cannot conclusively determine the causal relationship between shift work and elevated uric acid level because of the cross-sectional study design. Additional research on the mechanism responsible for the association between shift work and elevated uric acid level is required and the results of prospective cohort studies are needed. Second, the relationship of diet to serum uric acid level was not measured in our study. Third, this study targeted a specific occupational group. Further studies targeting the entire population of Korea will be required to examine the relationship between working conditions and uric acid level.

## Conclusion

In conclusion, we observed a higher prevalence of hyperuricemia in shift workers than among day workers.
